# Antioxidant and Antimicrobial Potential of Phenolic Metabolites from Traditionally Used Mediterranean Herbs and Spices

**DOI:** 10.3390/foods8110579

**Published:** 2019-11-15

**Authors:** Ivana Generalić Mekinić, Danijela Skroza, Ivica Ljubenkov, Višnja Katalinić, Vida Šimat

**Affiliations:** 1 Department of Food Technology and Biotechnology, Faculty of Chemistry and Technology, University of Split, Ruđera Boškovića 35, HR-21000 Split, Croatia; danci@ktf-split.hr (D.S.); visnja@ktf-split.hr (V.K.); 2 Department of Chemistry, Faculty of Science, University of Split, Ruđera Boškovića 33, HR-21000 Split, Croatia; iljubenk@pmfst.hr; 3 Department of Marine Studies, University of Split, Ruđera Boškovića 37, HR-21000 Split, Croatia; vida@unist.hr

**Keywords:** phenolic compounds, herbs, antioxidants, antimicrobials, HPLC, PCA

## Abstract

The phenolic extracts of fifteen Mediterranean medicinal plants, as well as their antioxidant and antimicrobial activities were investigated to grade their potential as additives in the food industry. Phenolic profiles of plant extracts were determined spectrophotometrically (total phenolics and phenolic subgroups) while individual compounds were identified using chromatographic assays. The biological activity of samples was determined using five antioxidant assays, while the antibacterial potential was determined against six foodborne pathogens (*Camplyobacter coli, Escherichia coli, Salmonela* Infantis, *Bacillus cereus*, *Listeria monocytogenes*, and *Staphylococcus aureus*). The results showed significant variations in phenolic profile of plants and consequently their biological activity. Bearberry contained the highest concentration of phenolics, was extremely rich in non-flavonoids and also had the highest amount of catechins that resulted with good reducing and free radical scavenging properties and low chelating activity. All extracts were not effective against tested microorganisms with Gram-positive bacteria being more sensitive (especially *S. aureus*). The most effective extracts were St. Johns wort against *S. aureus* with minimal inhibitory concentration (MIC) of 1.00 mg/mL), bay laurel and nettle against *B. cereus* (MICs of 1.67 mg/mL), and woodland strawberry against *L. monocytogenes* (MIC of 3.33 mg/mL).

## 1. Introduction

The positive biological effects and health benefits of herbs and spices have made them irreplaceable in traditional and folk medicine around for centuries. The World Health Organization is reporting that 80% of the populations in developing countries rely on the use of plant-based medicines for healthcare despite the development of modern medicine [1]. It has been estimated that global trade in medicinal plants is over US$100 billion per year with the annual growth rate of about 10%–15% [2,3]. The health promoting properties of herbs have been related to the presence of various groups of secondary metabolites, especially phytochemical constituents such as phenolic compounds with numerous positive biological activities [4]. The most important and widely investigated feature of phenolics is their protecting effect from damage caused by free radical induced oxidative stress, and this protection can cause delay and/or inhibition of various diseases and degenerative conditions such as cardiovascular diseases, atherosclerosis, cancer, central nervous system disorders, Parkinson’s and Alzheimer’s diseases, diabetes, respiratory, and autoimmune diseases [5,6]. Another major significance of phenolic compounds is their efficient antimicrobial activity allowing their use in treatment of different health disorders, diseases, and conditions caused by different microorganisms [7,8].

The application of herbs and spices in the food industry has also been well studied. The control of food quality and spoilage, affected by different factors such as physical, chemical, and microbiological, is essential for food preservation. Oxidation and microbiological spoilage are the major causes of food deterioration, and these processes compromise food safety and have negative effects on organoleptic properties and nutritional quality of food products. Safe, high-quality, and shelf-stable foods have become a challenge, thus finding natural additives that are able to delay or inhibit the oxidation and/or growth of microorganisms would contribute to development of sustainable solution for the food industry. In the last few decades there is a growing interest in natural compounds with strong biological activities and their application as preservatives for prolonging the shelf-life and improving the safety of perishable food products [9,10,11].

The countries of the Mediterranean coastal region use wild-growing plants and herbs in traditional medicine and nutrition. Due to low productivity of rocky soils and specifics of Mediterranean climate (hot summers with low rainfalls, and moderate temperatures and abundant rainfall the rest of the year) the Dalmatian karst is a native habitat for various herbs, some of which are cultivated in numerous countries worldwide [12]. The aim of this study was to investigate the phenolic potential of fifteen medicinal plants that have been used in Mediterranean folk medicine from ancient times, and to determine their antioxidant and antimicrobial activities. Additionally, the study aimed to find the correlation between their chemical composition of the plants and related biological capacity to get an insight into their biopreservative potential and potential health benefits.

## 2. Materials and Methods

### 2.1. General

Dry plant materials (bearberry, meadowsweet, woodland strawberry, yellow bedstraw, herb Robert, St. Johns wort, walnut, bay laurel, sweet basil, olive leaves, knotweed, winter savory, mountain germande, nettle, common speedwell) were produced by Suban (Samobor, Croatia) and purchased from a local herbal pharmacy. All used reagents, solvents, and standards were of adequate analytical grade and were obtained from Kemika (Zagreb, Croatia), Merck (Darmstadt, Germany), Oxoid (Hampshire, UK), Fluka (Buchs, Switzerland), Riedel-de Haen AG (Seelze, Germany), and Sigma-Aldrich GmbH (Steinheim, Germany).

Antimicrobial activity was screened against six bacterial strains, namely *Bacillus cereus* WSBC 10530 (clinical isolate), *Campylobacter coli* ATCC 33559 (pig feces isolate), *Escherichia coli* O157:H7 ŽM370 (clinical isolate), *Listeria monocytogenes* ŽM58, *Salmonella* Infantis ŽM9 (poultry meat isolate), *Staphylococcus aureus* ATCC 25923 (clinical isolate).

Spectrophotometric measurements were performed on a SPECORD 200 Plus, Edition 2010 (Analytik Jena AG, Jena, Germany) and Synergy HTX Multi-Mode Reader (BioTek Instruments, Inc., Winooski, VT, USA). The high-performance liquid chromatography (HPLC) system used was composed by a Varian 330 UV/Vis photodiode array detector, a ternary gradient liquid Pro Star 230 pump, a model 500 heater, Star 6.0 chromatography workstation (Varian Inc., Walnut Creek, CA, USA).

### 2.2. Plant Material and Extract Preparation

The extraction procedure was previously reported in Generalić Mekinić et al. [13]. Pulverised (1 min in high speed grinder) dry plant material (5 g) was extracted with a mixture of ethanol/water (250 mL, 80/20, v/v) at 60 °C for 60 min. After cooling, the samples were filtered and the residual tissue washed with solvent (3 × 10 mL). The extractions were performed in triplicate for each sample. After the extraction, the total polyphenol index of the extracts was determined according to the reference procedure described by González-Rodríguez et al. [14] and three extracts of the same plant material were combined and evaporated to the volume of 150 mL, centrifuged (2330× *g*, 5 min) and used for further analysis.

### 2.3. Phenolic Composition

#### 2.3.1. Total Phenolics, Non-Flavonoids, Flavonoids, Flavanol Monomers, and Proanthocyanidins

The total phenolic content in plant extracts was determined by the Folin–Ciocalteu method [15,16], non-flavonoids (NF) using the method reported by Kramling and Singleton [17] while the flavonoids (FLA) were calculated as the difference between TP and NF. The concentration of flavanol monomers and proanthocyanidins (FMP) was determined using the vanillin assay [15], while the amount of flavanol monomers (FM) was estimated using the *p*-dimethylaminocinnamaldehyde (DMACA) method [17,18]. The results for TP, NF, and FLA were expressed as mg gallic acid equivalents (mg GAE/g), for FMP as mg epicatechin equivalents (mg EE/g), and for FM as mg catechin equivalents (mg CE/g) per gram of dry plant material. 

#### 2.3.2. Individual Phenolic Compounds

The HPLC was used for identification and quantification of individual phenolic compounds as described in Generalić et al. [19]. Rosmarinic acid and flavonoids were separated on an Ultra Aqueous C18 column (250 × 4.6 mm, 5 mm; Restek; maintained at 30 °C), while monomeric acids were separated on a Zorbax Eclipse XDB-C18 column (250 × 4.6 mm, 5 mm; Agilent; maintained at 25 °C). The applied flow rate was 1.0 mL/min. The peaks of detected phenolics were identified by comparing their retention times and absorption spectra with those acquired for corresponding standards. Spiking was used to assist confirmation of the peak identity and the identified compounds were quantified using external standard calibration curves. The results are expressed in mg of compound per g of dry plant material (mg/g).

### 2.4. Antioxidant Activity

The reducing activity was measured as Ferric Reducing/Antioxidant Power (FRAP), using a method described by Benzie and Strain [20] and the results expressed in micromoles of Fe^2+^ per litre of extract (µM Fe^2+^).

DPPH (2,2-diphenyl-1-picrylhydrazyl) scavenging ability was measured according to the modified procedure of Von Gadow, Joubert, and Hansmann [21] described in detail by Katalinić et al. [17] and 2,2′-azinobis-(3-ethylbenzothiazoline-6-sulfonic acid) radical cation (ABTS) scavenging ability according to the procedure reported by Re et al. [22]. The final results were expressed as inhibitory concentrations (IC_50_), mg GAE/L of extract needed to reduce radicals by 50%.

The inhibitory activity of extracts on oscillations in the Briggs–Rauscher reaction system was estimated as the time (in minutes) before oscillations restart [19,23]. The analysed plant extracts were diluted to a total phenol concentration of 100 mg GAE/L.

The chelating potential was estimated using the colorimetric assay described by Yen, Duh, and Chuang [24]. The extract concentration (in mg GAE/L) that is effective in the chelation of metal ions by 50% (CHEL IC_50_) was calculated from the dose-response curve plotting between % of chelating activity and concentration.

### 2.5. Antimicrobial Activity

The *C. coli* was incubated micro-aerobically at 42 °C on Müeller Hinton broth (MHB) with 5% of defibrinated horse blood (Oxoid) while the other bacterial cultures were incubated aerobically at 37 °C on Müeller Hinton broth or agar (MHA). For the inoculum preparation cultures were incubated (at the same conditions) for 20 h on MHB and for antibacterial assays 1 mL of each inoculum was appropriately diluted with MHB to reach approximately 10^5^–10^6^ CFU/mL. Minimum inhibitory concentration (MIC) was detected by the broth microdilution method according to the procedure described by Klančnik et al. [25]. In order to reduce the ethanolic content in each well to the concentration that will not affect the tested microorganisms, the plant extracts were previously diluted to 40% (v/v) stock solutions in MHB. The MIC value is the lowest concentration where no metabolic activity was observed. The results are endorsed by three measurements and are expressed in mg of dry plant material per mL of growth medium (mg/mL).

### 2.6. Statistical Analysis

All analyses were carried out in triplicate and the data are given as the mean ± standard deviation, except the MIC values were the obtained values from all three measurements. STATISTICA (vision 13, StatSoft Inc, Tulsa, OK, USA) was used for data analysis. Pearson’s correlation coefficient (*p* < 0.05) was used for determination of the relations between the variables. The statistical differences between investigated parameters were determined by analysis of variance (one-way ANOVA) followed by a Fisher’s least significant difference test at a 95% confidence level. Principal component analysis (PCA) was used to establish relations between the studied variables.

## 3. Results and Discussion

The biological properties of herbs have been related to their ability to synthesize compounds that possess antioxidant and antimicrobial activity, with particular focus on phenolics that possess both of these characteristics [11]. Fifteen medicinal plants from twelve plant families that have been investigated in this study are listed in Table 1.

The results for plant extract phenolic content; share of total phenols (TP), flavonoids (FLA), non-flavonoids (NF), flavanol monomers (FM) and flavanol monomers and procyanidins (FMP), are shown in Table 2. Among plants, bearberry extract contained the highest concentration of TP while their concentration in all other extracts was significantly lower (from 71.2 mg GAE/g in yellow bedstraw to 347.8 mg GAE/g in meadowsweet). The bearberry extract was also extremely rich in NF (511.7 mg GAE/g), and this phenolic fraction was also dominant in mountain germander (90%), yellow bedstraw (87%), herb Robert (77%), and knotweed (73%). On the other hand, a higher share of FLA was detected in St. Johns wort (75%), bay laurel (75%), walnut (65%), and meadowsweet (61%) extracts. Bearberry, meadowsweet, St. Johns wort, walnut, bay laurel, and knotweed extracts contained high concentration of FM and FMP with the highest content of both subgroups detected in St. Johns wort, 56.2 mg EE/g and 12.8 mg CE/g, respectively. Katalinić et al. [26] reported a study on total phenolic and antioxidant activity of 70 medicinal plants of the Mediterranean area. Although different extraction procedures were used the highest phenolic content was detected in bearberry extract. Komes et al. [27] investigated the effect of extraction time and hydrolysis on the qualitative and quantitative content of phenolics and antioxidant activity of six traditionally used herbs, among which were olive and nettle. They also reported slight domination by the non-flavonoids in those extracts, as well as low content of FM and FMP. The domination of non-flavonoids in Lamiaceae plant extracts was confirmed due to the high content of phenolic acids; especially rosmarinic acid, that also was confirmed by this study [13,28,29,30]. Wojdylo et al. [31] also reported the domination of flavonoids in St. Johns wort (only 10% of total phenols were phenolic acids), and had similar content of FLA and NF in walnut extracts as in our study.

The individual phenolic compounds in plant extracts are shown in Table 3 and Table 4. Gallic acid was the most abundant hydroxybenzoic acid with highest concentrations detected in meadowsweet, woodland strawberry, and bearberry, 1.71, 1.03, and 0.98 mg/g, respectively. Among hydroxycinnamic acids, the most abundant was ferulic acid with highest concentrations found in woodland strawberry, while in the other two above mentioned extracts its presence was not confirmed. The dominant phenolic acid in bearberry extract was syringic acid (24.69 mg/g), in woodland strawberry it was vanillic acid (12.60 mg/g), and in all three extracts from Lamiaceae plants, sweet basil, winter savory, and mountain germander, it was rosmarinic acid. The results of qualitative analysis of phenolic compounds demonstrated that rosmarinic acid is the predominant phenolic compound in species of the Lamiaceae family [13,27]. Previous studies also reported the presence of caffeic acid in nettle (0.84 mg/g in hydrolised extract) [27], olive (0.003 mg/g) [32] and meadowsweet (4.2 mg/g) [33], St. Johns wort (2.3 mg/g), knotweed (0.21 mg/g) and walnut (1.5 mg/g) [31], *p*-coumaric (0.01 mg/g) and ferulic acid (0.02 mg/g) in nettle [27], St. Johns wort (0.09 mg/g) and walnut (0.35 mg/g) [31]. St. Johns wort contained the highest amounts of protocatechuic acid, *p*-hydroxybenzoic acid, and caffeic acid, while winter savory was rich in vanillic and syringic acid. Danila et al. [34] reported the presence of caffeic (0.6 mg/g), ferulic (8.5 mg/g), and coumaric acid (1.9 mg/g) in yellow bedstraw. Among flavonoids, rutin was not detected in only three extracts, namely knotweed, mountain germander, and common speedwell, and its highest concentration was detected in woodland strawberry (2.67 mg/g), while the lowest was found in winter savory (0.10 mg/g). Bearberry contained the highest amount of catechin, but also extracts of yellow bedstraw and St. Johns wort were rich in this compound, while detected concentrations of its epimer ranged from 0.10 mg/g in walnut extract to 0.49 mg/g in bearberry extract. The concentration of quercetin-4-glucoside in meadowsweet was 4.74 mg/g, and in accordance with data reported by Harbourne et al. [35]. Kaempferol was the only investigated flavonol detected only in four extracts, while among flavones, luteolin and apigenin, the highest concentrations were detected in winter savory extract. Wojdylo et al. [31] also detected quercetin and kaempferol in St. Johns wort (0.5 and 0.06 mg/g, respectively) and walnut extract (4.6 and 0.88 mg/g, respectively), while in their study the presence of investigated flavonoids in knotweed was not confirmed. Although in this study the identification of each phenolic compound was carried out by several methods, other techniques such as mass spectrometry should be applied for their proper confirmation.

**Figure 1:**
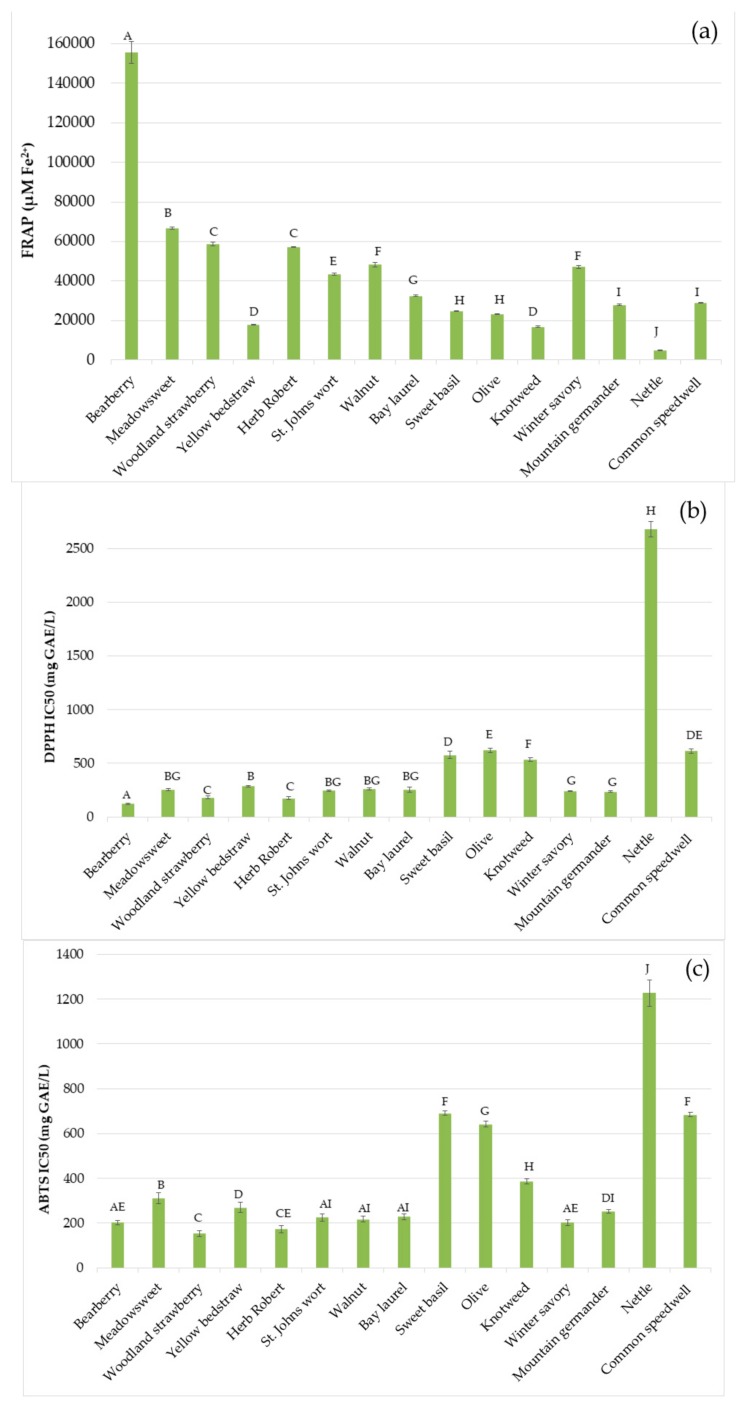

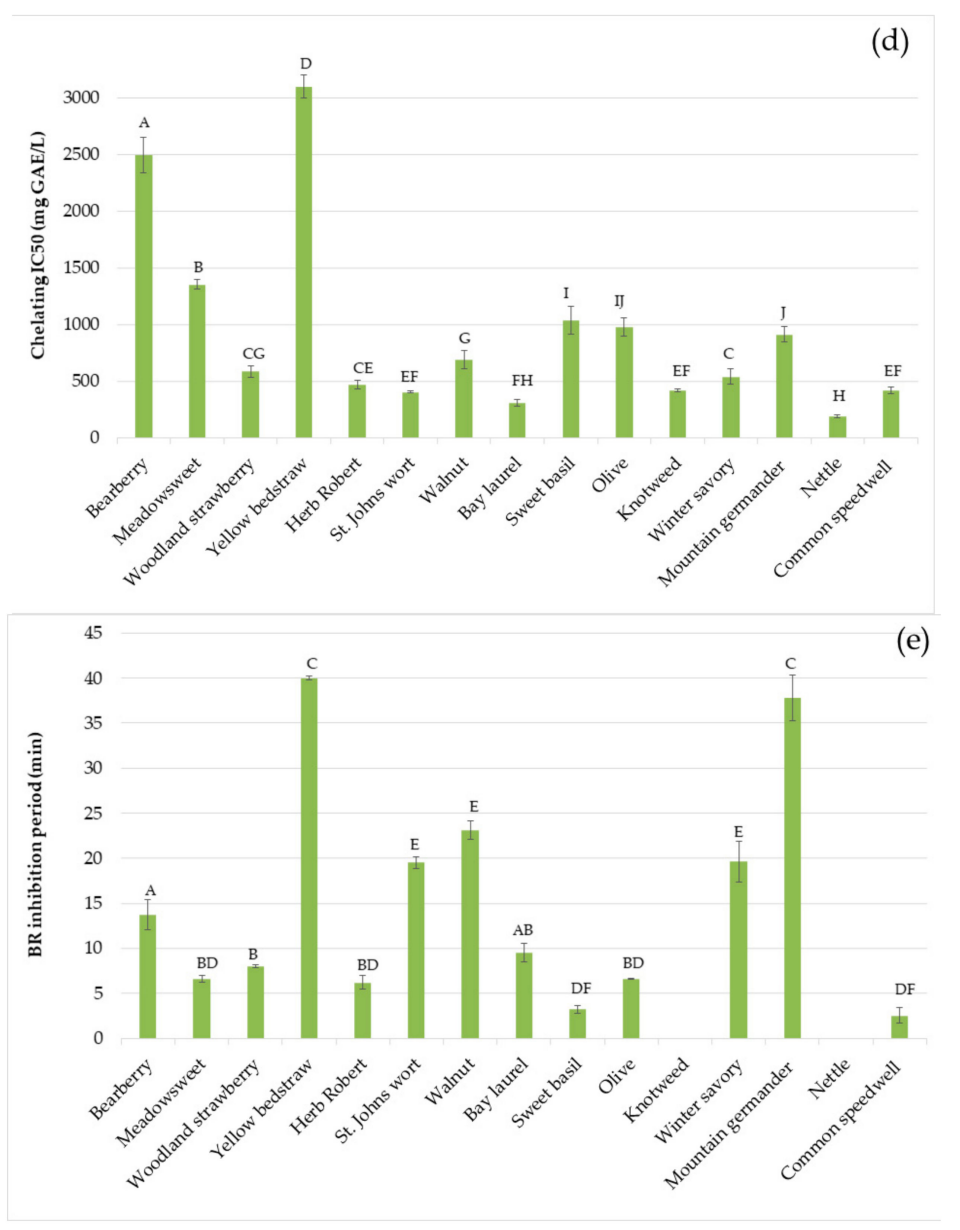
Antioxidant activity of plant extracts evaluated by (**a**) Ferric reducing antioxidant Power assay (FRAP), as scavenging activity against two free radicals: (**b**) 2,2-diphenyl-1-picril-hydrazyl (DPPH), (**c**) 2,2′-azinobis-(3-ethylbenzothiazoline-6-sulfonic acid) radical cation (ABTS), (**d**) chelating and (**e**) Briggs–Rauscher assay. Different letters (A–J) of above columns denote statistically significant difference (*p* < 0.05) in antioxidant activity of plant extracts.

The results of the antioxidant activity assessed using five methods are shown in Figure 1. The highest reducing activity detected as FRAP value was measured for bearberry extract and it was more than 2-fold higher than for the meadowsweet extract that showed the second highest reducing ability (66.6 mmol Fe^2+^). All other extracts showed good reducing ability (range from 16770 to 58750 µmol Fe^2+^) except nettle extract that provided an extremely low FRAP value. Free radical scavenging ability of this extract was also very weak and IC_50_ values were high, 2679 mg GAE/L for DPPH and 1227 mg GAE/L for ABTS, respectively. Bearberry extract showed the best DPPH scavenging activity while better activity was observed against the ABTS radical by woodland strawberry and Herb Robert.

Among all tested extracts, nettle extract showed best chelating activity (IC_50_ of 186 mg GAE/L), while the highest values chelating IC_50_ values were detected for yellow bedstraw (3100 mg GAE/L), bearberry (2492 mg GAE/L), meadowsweet (1352 mg GAE/L), and sweet basil (1035 mg GAE/L). Yellow bedstraw and mountain germander inhibited Briggs–Rauscher oscillating reactions for a longest period, 40 and 38 min, while extracts of knotweed and nettle did not show any activity using this method. Katalinić et al. [26] also reported extremely good reducing capacity of bearberry as well as significant linear correlation between total phenolic content and FRAP.

The antibacterial activities of investigated herbs against *E. coli*, *Salmonela* Infantis, *C. coli*, *B. cereus*, *S. aureus,* and *L. monocytogenes* are shown in Table 5. From the obtained results it can be seen that differences were found in the susceptibility of the bacterial strains used, especially as all extracts were not effective against all tested microorganisms, especially against *E. coli* and *Salmonela* Infantis (Table 3). Among investigated Gram-negative bacterial strains, all extracts were effective against *C. coli*, with the lowest MIC detected was for yellow bedstraw, St. Johns wort, and nettle. Gram-positive bacteria are more sensitive to antimicrobial agents because of their membrane structure so in this study lower MIC values were obtained for these species in comparison to Gram-negative ones. The most resistant organism was *E. coli*, while the most susceptible bacterial strain was *S. aureus*. The most effective extract against *S. aureus* was St. Johns wort (MIC value of 1.00 mg/mL), against *B. cereus* were bay laurel and nettle (with both MICs of 1.67 mg/mL), and against *L. monocytogenes* it was woodland strawberry (MIC of 3.33 mg /mL). The high content of flavonols and flavones in extracts that showed significant antimicrobial activity, especially against Gram-negative species, may be the reason for their antimicrobial properties [8]. If we compare these results with those obtained in our previous studies where we investigated the antimicrobial activity of Asteraceae plant extracts [36] it can be seen that extracts of common yarrow were more effective against *C. coli* while they had no effect on *E. coli* and *Salmonela* Infantis. Moreover, Asteraceae extracts were more efficient against *S. aureus*. Immortelle also provided better results against the other two Gram-positive species in comparison to the result obtained for extracts from this study. Furthermore, if we compare the results from Lamiaceae extracts with those already published [13] it was observed that winter savory had the lowest activity against *C. coli* among all tested extracts. The same observations could be noted for sweet basil which showed the lowest antibacterial activities against Gram-positive species, while the other two extracts, among all tested Lamiaceae plants, showed the highest activity against *S. aureus* and *B. cereus*. Abramovič et al. [37] reported results for effective antibacterial activities of basil against *Campylobacter jejuni*, *S. aureus*, and *L. monocytogenes* that correlate with the results of our study and confirm better activity of basil extracts against Gram-positive species.

**Figure 2:**
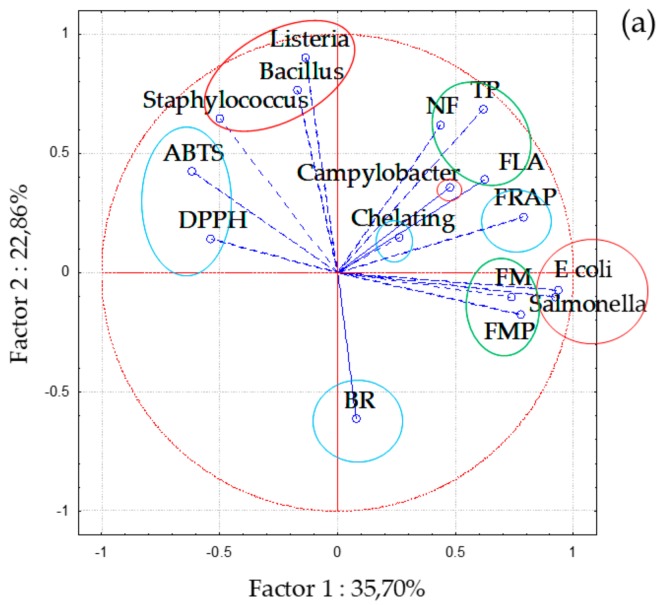

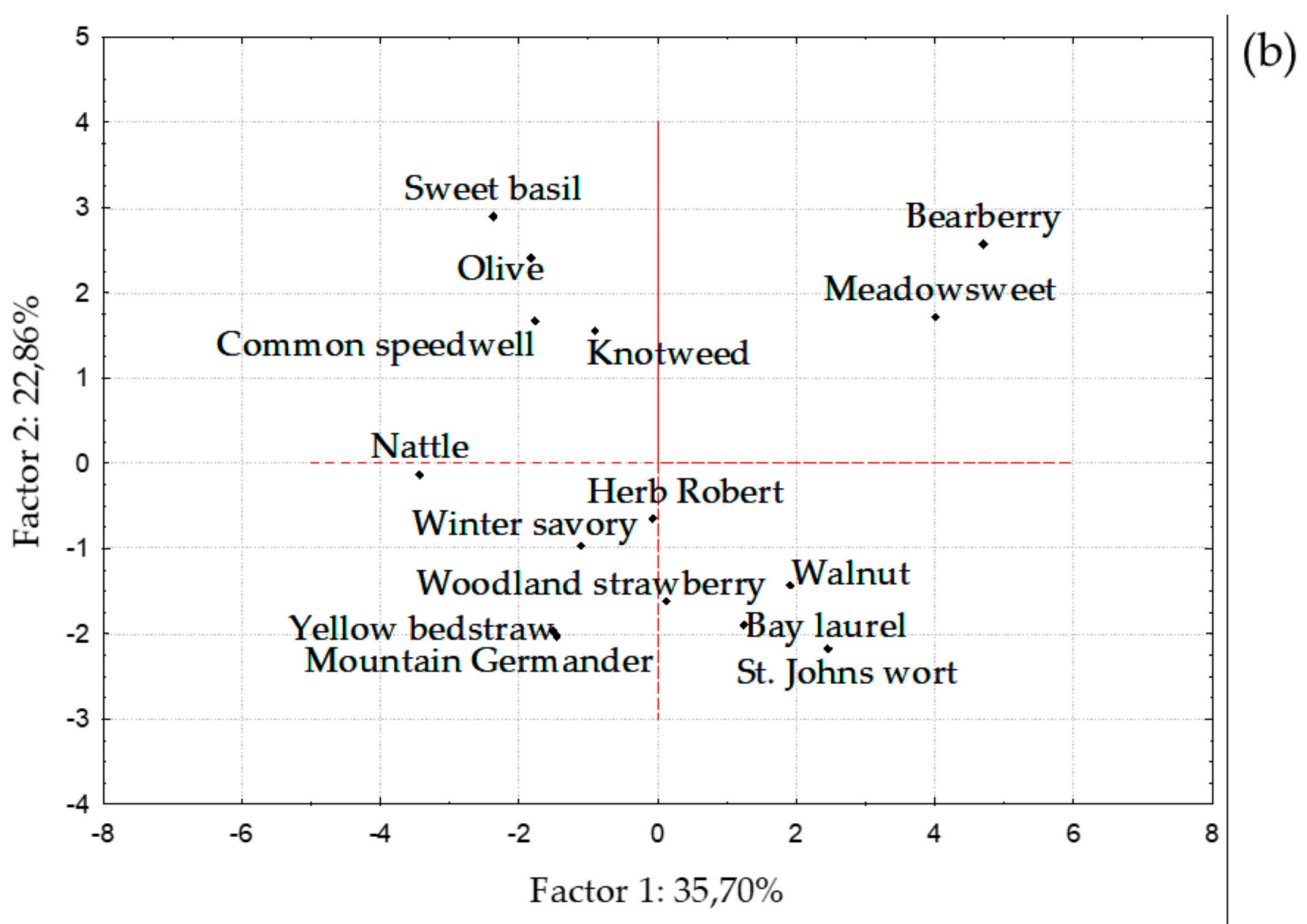
Principal component (PC) analysis of plant extracts based on PC1 and PC2 scores; loading plots of (**a**) investigated parameters, and (**b**) plant extracts.

A multivariate data analysis, the principal component analysis (PCA), was performed to compare and classify the investigated medicinal plants according to their chemical composition and antioxidant/antibacterial capacity. As shown in Figure 2, PCA calculation showed that the two first principal components (PC1 and PC2) are responsible for 58.56% of variability among the plant extracts. The score plot of PCA showed the projection and clear separation of samples in the multivariate space of the first two PCs, and according to the factor scores, the investigated are positioned in the two-dimensional space, with some obvious groupings (Figure 2a). The major phenolic groups (TP, FLA, NF) are positioned in the upper right quadrant, while less abundance sub-groups of FM and FMP are positioned below. This part of the loading plot also contains antioxidant activity parameters. FRAP method is positioned on the right side of the loading plot near to TP, FLA, and NF as this antioxidant assay is characterized by a simple electron-transfer and has the reducing mechanism similar to that characteristic for Folin–Ciocalteu assay. The second antioxidant method that is based on a special mechanism of chelating ionic molecules has been positioned near the center of the loading plot, while the BR method which detects the combined activity of antioxidants (antiradical, reducing, and chelating) is the only antioxidant method positioned in the lower part of the loading plot. Furthermore, the remaining two antioxidant assays, DPPH and ABTS which describe free radical scavenging activities of the samples are positioned on the opposite left side. Clear separation could also be seen for tested bacterial strains; Gram-positive strains that are positioned on the top of the upper left quadrant and Gram-negative ones positioned on the right side of the loading plot. Additionally, a small exception was observed for the results obtained against *C. coli* that also positioned this strain away from the other Gram-negative species.

Figure 2b presents the distribution of plant extracts in the two-dimensional space where also separation of extracts could be noted. Extracts were separated along both axes based on their phenolic content. Bearberry and meadowsweet extracts which were characterized with the highest total phenolic content and reducing activity are the only two extracts positioned in the upper right quadrant of the loading plot. These two extracts were also extremely effective against all investigated foodborne pathogens with the lowest MICs detected for bearberry, especially against Gram-positive species. The extracts of walnut, bay laurel, and St. Johns wort, which contain high amount of FM and FMP are separated in the lower right quadrant. These extracts also showed the antibacterial activity against tested microorganisms, while other extracts generally were not effective against *E. coli* and *Salmonella* Infantis. The extracts positioned in the upper left quadrant had low free radical scavenging activity, while yellow bedstraw and Mountain Germander which were rich in NF and showed low antimicrobial activity against the Gram-positive bacterial strains were positioned in the lower left quadrant. The PCA results are in accordance with those obtained by analysis of variance.

## 4. Conclusions

The results contribute to the search for sustainable, innovative, and natural food preservatives with biopreservative potential such as phenolic metabolites from traditionally used Croatian herbs and spices. Although the majority of herbs are widely known and already well investigated there are numerous factors that affect synthesis and accumulation of plant metabolites, such as phenolics, that complicates the comparison of the results from different studies, such as plant vegetation (collection) period, investigated tissue, used extraction solvent and protocol, temperature, rainfall, light intensity, soil composition, and nutrients availability, interactions with pathogens, etc. Although variations among the results were expected also due to the diversity of investigated plant species, the detected differences in antioxidant and antibacterial activities between samples were a consequence not only of variations in total phenolics but also in differences of the amount of individual phenolic compounds that should be taken into account in future studies.

## Figures and Tables

**Table 1:** List of investigated herbs.

Common Name	Pharmacopeial Name	Latin Name	Family	Plant Part
Bearberry	Uvae ursi folium	*Arctostaphylos uva-ursi* (L.) *spreng.*	Ericaceae	leaves
Meadowsweet	Filipendulae ulmariae herba	*Filipendula ulmaria* L.	Rosaceae	herb
Woodland strawberry	Fragariae folium	*Fragaria vesca* L.	Rosaceae	leaves
Yellow bedstraw	Galii veri herba	*Galium verum* L.	Rubiaceae	herb
Herb Robert	Geranii robertiani herba	*Geranium robertianum* L.	Geraniaceae	herb
St. Johns wort	Hyperici herba	*Hypericum perforatum* L.	Hypericiceae	herb
Walnut	Juglandis folium	*Juglans regia* L.	Juglandaceae	leaves
Bay laurel	Lauri folium	*Laurus nobilis* L.	Laureaceae	leaves
Sweet basil	Basilici herba	*Ocimum basilicum* L.	Lamiaceae	herb
Olive	Oleae folium	*Olea europea* L.	Oleaceae	leaves
Knotweed	Polygonum herba	*Polygonum aviculare* L.	Polygonaceae	herb
Winter savory	Satureja montana herba	*Satureja montana* L.	Lamiaceae	herb
Mountain germander	Teucrii montani herba	*Teucrium montanum* L.	Lamiaceae	herb
Nettle	Urticae folium	*Urtica dioica* L.	Urticaceae	leaves
Common speedwell	Veronicae herba	*Veronica officinalis* L.	Plantaginaceae	herb

**Table 2:** Total phenolics (TP), flavonoids (FLA), non-flavonoids (NF), flavanol monomers (FM), and flavanol monomers and procijanidins (FMP) in investigated plants.

Plant Extract	TP (mg GAE/g)	FLA (mg GAE/g)	NF (mg GAE/g)	FM (mg EE/g)	FMP (mg CE/g)
Bearberry	607.5 ± 2.1 ^A^	97.0 ± 1.4 ^A^	511.7 ± 1.1 ^A^	38.6 ± 0.9 ^A^	5.8 ± 0.3 ^A^
Meadowsweet	347.8 ± 3.6 ^B^	214.0 ± 3.4 ^B^	133.8 ± 0.3^B^	39.6 ± 0.4 ^B^	11.4 ± 0.3 ^B^
Woodland strawberry	134.1 ± 1.3 ^C^	45.1 ± 1.2 ^C^	89.0 ± 0.2 ^C^	5.4 ± 0.6 ^C^	3.1 ± 0.1 ^C^
Yellow bedstraw	71.2 ± 1.2 ^D^	8.9 ± 0.8 ^D^	62.2 ± 0.5 ^D^	16.7 ± 0.2 ^D^	0.0 ± 0.0 ^D^
Herb Robert	119.6 ± 0.5 ^E^	26.7 ± 0.7 ^E^	92.6 ± 0.8 ^E^	1.1 ± 0.2 ^EH^	0.2 ± 0.0 ^D^
St. Johns wort	145.3 ± 0.7 ^F^	109.4 ± 0.4 ^F^	35.8 ± 0.3 ^F^	56.2 ± 1.0 ^F^	12.8 ± 0.0 ^E^
Walnut	169.0 ± 0.5 ^G^	109.7 ± 0.3 ^F^	59.3 ± 0.3 ^G^	43.0 ± 0.6 ^G^	6.5 ± 0.0 ^F^
Bay laurel	148.3 ± 0.6 ^H^	110.5 ± 0.1 ^F^	37.7 ± 0.7 ^F^	39.5 ± 0.7 ^AB^	12.4 ± 0.1 ^G^
Sweet basil	212.4 ± 1.1 ^I^	94.0 ± 0.9 ^A^	118.4 ± 0.6 ^H^	1.9 ± 0.1 ^E^	0.0 ± 0.0 ^D^
Olive	223.0 ± 2.0 ^J^	77.6 ± 4.4 ^G^	143.4 ± 2.4 ^I^	1.1 ± 0.1 ^EH^	0.0 ± 0.0 ^D^
Knotweed	207.0 ± 1.2 ^K^	54.7 ± 4.1 ^H^	150.4 ± 3.1 ^J^	46.2 ± 1.2 ^I^	5.4 ± 0.2 ^H^
Winter savory	109.8 ± 0.7 ^L^	52.2 ± 0.5 ^H^	57.6 ± 0.2 ^G^	0.7 ± 0.1 ^H^	0.0 ± 0.0 ^D^
Mountain Germander	89.4 ± 2.0 ^M^	8.5 ± 2.6 ^D^	80.9 ± 3.3 ^K^	8.8 ± 0.2 ^J^	0.0 ± 0.0 ^D^
Nettle	150.6 ± 0.2 ^H^	152.4 ± 0.6 ^H^	98.2 ± 0.7 ^L^	1.0 ± 0.0 ^EH^	0.1 ± 0.0 ^D^
Common speedwell	227.9 ± 0.9 ^N^	96.6 ± 1.1 ^A^	129.3 ± 0.2 ^M^	10.2 ± 0.3 ^K^	0.1 ± 0.0 ^D^

GAE- gallic acid equivalents, EE- epicatechin equivalents, CE- catechin equivalents. Different letters (A–N) in superscript in the same column denote statistically significant difference (*p* < 0.05) in content of phenolics in plant extracts.

**Table 3:** Profile of the phenolic acids (mg/g) in investigated herb extracts.

Plant Extract	Hydroxybenzoic Acid Derivates						Hydroxycinnamic Acid Derivates					
	Gallic Acid	Protocatechuic Acid	*p*-hydroxyben. Acid	*m*-hydroxyben. Acid	Vanillic Acid	Syringic Acid	Caffeic Acid	*p*-coumaric Acid	*o*-coumaric Acid	*t*-ferulic Acid	Cinnamic Acid	Rosmarinic Acid
Bearberry	0.98 ± 0.01^A^	n.d.	0.89 ± 0.10 ^A^	n.d.	n.d.	24.69 ± 0.08 ^A^	n.d.	n.d.	n.d.	n.d.	0.08 ± 0.00 ^A^	n.d.
Meadowsweet	1.71 ± 0.07 ^B^	0.34 ± 0.02 ^A^	1.22 ± 0.07 ^B^	1.69 ± 0.46 ^A^	n.d.	n.d.	n.d.	0.35 ± 0.00 ^A^	n.d.	n.d.	0.18 ± 0.08 ^B^	n.d.
Woodland strawberry	1.03 ± 0.03 ^C^	n.d.	1.24 ± 0.03 ^B^	1.02 ± 0.03 ^B^	12.60 ± 0.04 ^A^	0.42 ± 0.00 ^B^	1.72 ± 0.04 ^A^	0.63 ± 0.01 ^B^	0.76 ± 0.01 ^A^	0.57 ± 0.13 ^A^	1.69 ± 0.01 ^C^	n.d.
Yellow bedstraw	0.02 ± 0.00 ^D^	0.11 ± 0.00 ^B^	0.30 ± 0.02 ^C^	n.d.	n.d.	0.06 ± 0.00 ^C^	0.08 ± 0.00 ^B^	0.04 ± 0.00 ^C^	n.d.	0.04 ± 0.00 ^E^	0.04 ± 0.00 ^D^	n.d.
Herb Robert	0.20 ± 0.01 ^E^	n.d.	n.d.	n.d.	n.d.	n.d.	n.d.	n.d.	n.d.	n.d.	n.d.	n.d.
St. Johns wort	0.02 ± 0.00 ^D^	0.52 ± 0.01 ^C^	3.66 ± 0.07 ^D^	n.d.	n.d.	0.08 ± 0.01 ^C^	2.41 ± 0.20 ^C^	n.d.	n.d.	0.18 ± 0.00 ^BD^	0.14 ± 0.00 ^EF^	n.d.
Walnut	0.02 ± 0.00 ^D^	0.37 ± 0.01 ^A^	n.d.	n.d.	n.d.	0.63 ± 0.02 ^D^	0.21 ± 0.01 ^D^	0.19 ± 0.01 ^D^	n.d.	0.13 ± 0.01 ^BC^	0.69 ± 0.01	n.d.
Bay laurel	0.20 ± 0.08 ^E^	0.34 ± 0.18 ^A^	0.43 ± 0.19 ^E^	n.d.	n.d.	n.d.	n.d.	0.11 ± 0.05 ^E^	n.d.	0.19 ± 0.10 ^BD^	0.07 ± 0.04 ^A^	n.d.
Sweet basil	0.02 ± 0.00 ^D^	n.d.	n.d.	n.d.	n.d.	n.d.	n.d.	n.d.	n.d.	n.d.	n.d.	18.38 ± 0.44 ^A^
Olive	0.04 ± 0.00 ^D^	n.d.	n.d.	n.d.	n.d.	0.13 ± 0.00 ^E^	0.35 ± 0.01 ^E^	0.42 ± 0.01 ^F^	n.d.	0.06 ± 0.01 ^CE^	0.12 ± 0.00 ^E^	n.d.
Knotweed	n.d.	n.d.	n.d.	n.d.	0.54 ± 0.02 ^B^	0.61 ± 0.00 ^D^	n.d.	n.d.	0.10 ± 0.00 ^B^	0.52 ± 0.02 ^A^	0.16 ± 0.01 ^BF^	n.d.
Winter savory	0.02 ± 0.00 ^D^	0.04 ± 0.01 ^B^	0.18 ± 0.01 ^F^	n.d.	0.82 ± 0.01 ^C^	1.20 ± 0.02 ^F^	0.33 ± 0.3 ^EF^	n.d.	0.15 ± 0.01 ^C^	0.04 ± 0.01 ^E^	n.d.	25.15 ± 0.47 ^B^
Mountain Germander	n.d.	0.06 ± 0.01 ^B^	0.24 ± 0.00 ^CF^	n.d.	1.05 ± 0.02 ^D^	0.13 ± 0.01 ^E^	n.d.	1.88 ± 0.00 ^G^	n.d.	0.23 ± 0.0 ^D^	n.d.	26.78 ± 0.38 ^C^
Nettle	0.02 ± 0.00 ^D^	n.d.	n.d.	0.22 ± 0.02 ^C^	n.d.	n.d.	1.64 ± 0.01 ^A^	0.03 ± 0.01 ^C^	n.d.	0.05 ± 0.00 ^E^	n.d.	n.d.
Common speedwell	0.18 ± 0.00 ^E^	0.20 ± 0.00 ^D^	n.d.	n.d.	0.93 ± 0.01 ^E^	n.d.	0.25 ± 0.02 ^DF^	0.03 ± 0.00 ^C^	0.13 ± 0.01 ^D^	0.43 ± 0.01 ^F^	0.03 ± 0.00 ^D^	n.d.

n.d.—not detected. Different letters (A–F) in superscript in the same column denote statistically significant difference (*p* < 0.05) in content of phenolic acids in plant extracts.

**Table 4:** Profile of the flavonoids (mg/g) in investigated herb extracts.

Plant Extract	Catechin	Epicatechin	Quercetine	Quercetin-4-glucoside	Rutin	Kaempferol	Luteolin	Apigenin
Bearberry	1.81 ± 0.08 ^A^	0.49 ± 0.04 ^A^	n.d.	n.d.	1.58 ± 0.05 ^A^	n.d.	n.d.	n.d.
Meadowsweet	0.70 ± 0.03 ^B^	n.d.	n.d.	4.74 ± 0.04 ^A^	0.68 ± 0.08 ^B^	n.d.	n.d.	n.d.
Woodland strawberry	0.20 ± 0.00 ^C^	n.d.	n.d.	n.d.	2.67 ± 0.01 ^C^	n.d.	n.d.	n.d.
Yellow bedstraw	1.41 ± 0.11 ^D^	0.11 ± 0.01 ^B^	0.32 ± 0,01 ^A^	n.d.	2.49 ± 0.04 ^D^	0.13 ± 0.00 ^A^	n.d.	n.d.
Herb Robert	n.d.	0.45 ± 0.04 ^C^	n.d.	0.81 ± 0.08 ^B^	2.13 ± 0.10 ^E^	n.d.	n.d.	n.d.
St. Johns wort	1.37 ± 0.05 ^D^	0.28 ± 0.00 ^D^	0.29 ± 0.01 ^B^	n.d.	2.39 ± 0.01 ^F^	0.13 ± 0.00 ^A^	n.d.	0.02 ± 0.00 ^A^
Walnut	0.64 ± 0.01 ^B^	0.10 ± 0.01 ^B^	0.20 ± 0.00 ^C^	n.d.	0.36 ± 0.04 ^G^	n.d.	0.30 ± 0.01 ^A^	0.04 ± 0.00 ^B^
Bay laurel	n.d.	0.26 ± 0.01 ^D^	n.d.	n.d.	1.05 ± 0.09 ^H^	n.d.	n.d.	n.d.
Sweet basil	n.d.	n.d.	0.04 ± 0.00 ^D^	0.94 ± 0.03 ^C^	0.70 ± 0.03 ^B^	n.d.	n.d.	n.d.
Olive	n.d.	n.d.	n.d.	n.d.	0.30 ± 0.00 ^GI^	n.d.	n.d.	n.d.
Knotweed	n.d.	n.d.	n.d.	1.59 ± 0.04 ^D^	n.d.	n.d.	n.d.	n.d.
Winter savory	n.d.	0.22 ± 0.02 ^E^	n.d.	n.d.	0.10 ± 0.01 ^J^	0.02 ± 0.00 ^B^	0.57 ± 0.02 ^B^	0.12 ± 0.01 ^C^
Mountain Germander	0.05 ± 0.00 ^E^	n.d.	n.d.	0.81 ± 0.08 ^B^	n.d.	n.d.	n.d.	n.d.
Nettle	0.86 ± 0.04 ^F^	n.d.	n.d.	n.d.	0.27 ± 0.02 ^I^	n.d.	0.15 ± 0.02 ^C^	0.05 ± 0.00 ^B^
Common speedwell	n.d.	n.d.	n.d.	n.d.	n.d.	0.02 ± 0.00 ^B^	n.d.	n.d.

n.d.—not detected. Different letters (A–J) in superscript in the same column denote statistically significant difference (*p* < 0.05) in content of flavonoids in plant extracts.

**Table 5:** Antibacterial activity of plant extracts expressed as minimal inhibitory concentration (MIC).

Plant Extract	MIC (mg/mL)					
	*Escherichia coli*	*Salmonella* Infantis	*Campylobacter coli*	*Staphylococcus aureus*	*Bacillus cereus*	*Listeria monocytogenes*
Bearberry	1.67	1.67	0.83	0.35	1.67	1.67
Meadowsweet	3.33	2.78	6.67	1.39	1.67	3.33
Woodland strawberry	3.33	3.33	1.67	1.24	3.33	3.33
Yellow bedstraw	n.d.	n.d.	1.67	8.08	6.66	6.66
Herb Robert	5.55	5.55	3.33	2.77	6.66	6.66
St. Johns wort	6.66	6.66	0.83	1.00	3.33	3.33
Walnut	5.24	5.24	3.33	1.70	3.33	3.33
Bay laurel	3.33	3.33	1.67	1.39	1.67	3.33
Sweet basil	n.d.	n.d.	1.67	5.56	6.67	6.67
Olive	n.d.	n.d.	3.33	2.78	6.67	6.67
Knotweed	n.d.	n.d.	1.67	2.78	6.66	6.66
Winter savory	n.d.	n.d.	6.66	2.44	6.66	6.66
Mountain Germander	n.d.	n.d.	3.33	3.07	6.66	6.66
Nettle	n.d.	n.d.	0.83	2.78	1.67	3.33
Common speedwell	n.d.	n.d.	0.83	2.78	3.33	6.67

n.d.—not determinated as the MIC value has not been detected by the maximal tested concentration.
